# Runx2 and Polycystins in Bone Mechanotransduction: Challenges for Therapeutic Opportunities

**DOI:** 10.3390/ijms25105291

**Published:** 2024-05-13

**Authors:** Antonios N. Gargalionis, Christos Adamopoulos, Christos T. Vottis, Athanasios G. Papavassiliou, Efthimia K. Basdra

**Affiliations:** 1 Laboratory of Clinical Biochemistry, Medical School, National and Kapodistrian University of Athens, ‘Attikon’ University General Hospital, 12462 Athens, Greece; agargal@med.uoa.gr; 2 Department of Biological Chemistry, Medical School, National and Kapodistrian University of Athens, 11527 Athens, Greece; cadamop@med.uoa.gr (C.A.); papavas@med.uoa.gr (A.G.P.); 3 Department of Oncological Sciences, Icahn School of Medicine at Mount Sinai, New York, NY 10029, USA; 4 First Department of Orthopedics, Medical School, National and Kapodistrian University of Athens, ‘Attikon’ University General Hospital, 12462 Athens, Greece; chvottis@gmail.com

**Keywords:** Runx2, polycystin-1, polycystin-2, mechanotransduction, osteoporosis, bone remodeling, YAP, TAZ

## Abstract

Bone mechanotransduction is a critical process during skeletal development in embryogenesis and organogenesis. At the same time, the type and level of mechanical loading regulates bone remodeling throughout the adult life. The aberrant mechanosensing of bone cells has been implicated in the development and progression of bone loss disorders, but also in the bone-specific aspect of other clinical entities, such as the tumorigenesis of solid organs. Novel treatment options have come into sight that exploit the mechanosensitivity of osteoblasts, osteocytes, and chondrocytes to achieve efficient bone regeneration. In this regard, runt-related transcription factor 2 (Runx2) has emerged as a chief skeletal-specific molecule of differentiation, which is prominent to induction by mechanical stimuli. Polycystins represent a family of mechanosensitive proteins that interact with Runx2 in mechano-induced signaling cascades and foster the regulation of alternative effectors of mechanotransuction. In the present narrative review, we employed a PubMed search to extract the literature concerning Runx2, polycystins, and their association from 2000 to March 2024. The keywords stated below were used for the article search. We discuss recent advances regarding the implication of Runx2 and polycystins in bone remodeling and regeneration and elaborate on the targeting strategies that may potentially be applied for the treatment of patients with bone loss diseases.

## 1. Introduction

Bone tissue continuously supports body movements and weight-bearing structures and is subjected to several environmental forces. It has been established that alterations of the mechanical forces imposed on the body evoke clinical manifestations, which vary from bone strengthening, because of long-term high intensity exercise, to bone loss and disuse osteoporosis, as a result of a minimal application of mechanical strain during paralysis or bed stay for a long period [1,2]. In this paper, we critically discuss the current findings regarding two key players of bone response to mechanical forces, Runt-related transcription factor 2 (Runx2) transcription factor and the major representatives of the polycystin family of proteins. It is critical to elucidate the implicated mechanisms of bone physiology and pathophysiology, as well as to highlight the specific mediators of these mechanisms, which will offer potential therapeutic targeting for bone diseases. The aim of this narrative review is to summarize and discuss data regarding these two key-drivers of mechano-induced signaling in bone cells, thereby providing further thoughts for the translational exploitation of bone mechanotransduction.

## 2. Molecular Mechanisms of Bone Mechanotransduction

The differentiation of the mechanical stimuli applied define the lifelong process of bone remodeling, which ensures the proper volume of bone formation and bone resorption according to environmental cues [2]. Bone remodeling is a dynamic process that is characterized by the notion that bone density changes as a response to mechanical forces applied on bone tissue. Bone remodeling involves osteogenic and osteolytic activities, meaning the processes of bone formation and bone loss, respectively. These are coordinated by osteoblasts, osteoclasts, osteocytes, and bone-lining cells [3]. Bone formation is facilitated by mechanical stresses and controls the production of protective or proinflammatory cytokines and the expression and activity of extracellular matrix (ECM) proteins, enzymes, and transcription factors. Furthermore, the mechanical stress generated by physical exercise can regulate hormone levels, such as those of parathormone (PTH), estrogen, and glucocorticoids, which are known to affect the anabolic/catabolic balance of bone homeostasis [3,4,5,6]. These mechanisms have been highlighted as part of mechanobiology, which is a multidisciplinary scientific field that studies the biological properties affected by cellular mechanical forces. To this end, deciphering the underpinning mechanisms of bone mechanobiology opens new therapeutic avenues towards bone regeneration following bone loss maladies or injuries.

Mechanotransduction is defined as the biochemical process through which cells respond to mechanical forces originating from the cell’s environment or the cell’s interior. Cells and subcellular structures are subjected to endogenous forces through cytoskeletal cell contractility and stresses applied from the extracellular environment. External forces include shear stress and hydrostatic pressure, which are observed when cells are accessible in fluid-filled lumens. Adjacent cells and tissues experience compression and tension, but also mechanical stimuli generated from ECM stiffness [7]. During bone cells’ differentiation, mechanical cyclical stretching induces bone and adipose tissues differentiation, fluid shear stress triggers the proliferation of osteoblasts, and compressive forces play an important role in osteoclasts’ formation. These forces demonstrate different thresholds and ranges that are required for regulating bone homeostasis [8]. It has been proven that cells transmit signals in a range from less than 1 millisecond (ms) to 10 ms, depending on the architecture of the cytoskeleton, cell shape, and density of mechanically induced channels. They can also detect mechanical energy within a wide range [9]. The biochemical signals generated by mechanical stimuli affect several cellular functions, such as gene expression, proliferation, differentiation, migration, apoptosis, and protein synthesis, ultimately regulating organ development, homeostasis, and skeletal regeneration [10,11].

**Figure 1:**
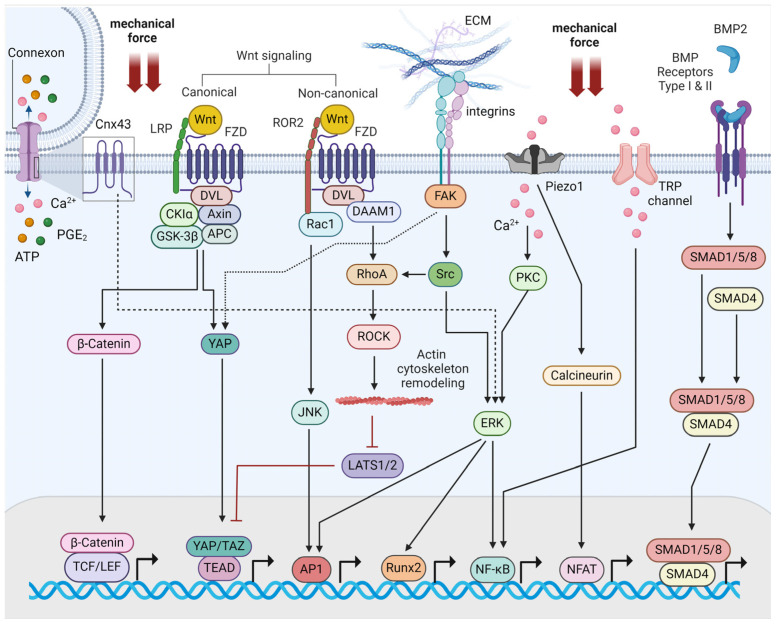
Schematic representation of bone mechanotransduction signaling pathways. AP1, activator protein-1; BMP2, bone morphogenetic protein 2; CKIα, casein kinase I alpha; Cnx43, connexin 43; DAAM1, disheveled-associated activator of morphogenesis 1; DVL, disheveled; ECM, extracellular matrix; ERK, extracellular signal-regulated kinase; FAK, focal adhesion kinase; FZD, frizzled; GSK-3*β*, glycogen synthase kinase-3 beta; JNK, c-Jun N-terminal kinase; LATS 1/2, large tumor suppressor kinase 1/2; LRP, low-density lipoprotein receptor-related protein; NFAT, nuclear factor of activated T cells; NF-κB, nuclear factor kappa light-chain enhancer of activated B cells; PKC, protein kinase C; PGE2, prostaglandin E2; ROCK, Rho-associated protein kinase; TAZ, transcriptional coactivator with PDZ-binding motif; TEAD, TEA domain family member 1; TCF/LEF, T-cell factor/lymphoid enhancer factor; TRP, transient receptor potential; YAP, yes-associated protein. This figure was created using the tools provided by BioRender.com (accessed on 24 April 2024).

The mechanotransduction of bone cells is mediated by mechanosensing structures, which are proteins that have the capacity to perceive mechanical forces. These are proteins of the ECM, focal adhesion complexes, mechanosensitive ion channels, apical membrane protrusions termed primary cilia, and membrane G protein-coupled receptors (GPCRs) [2,3]. The vital mechanosensitive proteins that are engaged in these structures are focal adhesion kinase (FAK) and Src kinase. Integrins represent a critical family of mechanosensors that become activated upon fluid flow stress and induce the opening of connexin 43 (Cx43) to release bone synthesis molecules, such as prostaglandin E2 (PGE2) [12]. Piezo1 and transient receptor potential (TRP) calcium ion channels mediate mechanical cues by activating the respective bone differentiating transcription factors [13]. Additional membrane-bound proteins trigger downstream signaling cascades involving the well-established effectors of signal transduction, such as Wnt/*β*-catenin pathway. In the canonical Wnt pathway, Wnt proteins bind to low-density lipoprotein receptor-related protein (LRP) and frizzled (FZD) co-receptors upon mechanical loading. This event ultimately leads to the accumulation of *β*-catenin to the nucleus through the involvement of several, closely regulated cytoplasmic proteins. *β*-catenin finally interacts with T-cell factor/lymphoid enhancer factor (TCF/LEF) transcription factors to regulate gene transcription [14]. Wnt proteins and LRP6 co-receptor can also activate yes-associated protein (YAP) and transcriptional coactivator with PDZ-binding motif (TAZ) transcription factors [15,16]. In the non-canonical pathway, the binding of Wnt proteins to FZD and ROR2 co-receptors activates downstream protein molecules, such as RhoA/Rho-associated protein kinase (ROCK) to trigger rearrangements of the cytoskeleton and activate c-jun NH_2_-terminal kinase (JNK) and activator protein1 (AP-1) transcription factors [17]. Furthermore, bone morphogenetic protein 2 (BMP2) binds to BMP receptors and regulates osteoblast and osteoclast homeostasis via a series of proteins from the Smad family of transcription factors [18]. Finally, YAP and TAZ transcriptional cofactors emerge as key mechanosensitive transcription factors in bone cells (Figure 1) [2,3,19]. YAP and TAZ are the final effectors of the Hippo signal transduction pathway that regulates mesenchymal lineage differentiation. YAP/TAZ are controlled by extracellular mechanical stimuli via nuclear shuffling that depends on cytoskeletal proteins. This happens without the interference of standard Hippo signaling. They integrate mechanical inputs and, especially, TAZ can translocate to the nucleus, induce the activation of Runx2, and enhance osteoblast differentiation [20].

## 3. Runx2 as an Effector of Bone Mechanotransduction

Runx2 belongs to the wider family of Runx transcription factors, which bind to the promoters of genes that regulate bone physiology. This family was described thirty years ago and includes Runx1, Runx2, and Runx3 members. The Runx family of transcription factors regulate various physiological processes, such as embryonic development, cell proliferation, differentiation, and cell lineage commitment [21]. Runx1/acute myeloid leukemia 1 (AML1) is lineage-specific for the differentiation of hematopoietic cells; Runx3/AML2 is specific for the differentiation of neuronal, gastrointestinal, and bone tissues; and Runx2/AML3 is specific for osteogenic cell differentiation, which refers to the process by which bone marrow stem cells transform into osteoblasts. The domain termed ‘runt’ is a DNA-binding domain of 128 amino acids and is the common structural feature of the family [22]. Runx2 is an essential transcriptional effector during the differentiation, maturation, and proper functioning of osteoblasts, chondrocytes, and mesenchymal stem cells [23,24].

The post-translational modifications of Runx2 fine-tune the function, stabilization, and localization of the protein. They include acetylation, interaction with chromatin modulators, phosphorylation, methylation, glycosylation, sumoylation, and ubiquitination. Whether phosphorylation upregulates or downregulates Runx2 depends on the residues phosphorylated. For example, the phosphorylation/activation of Runx2 occurs at serine residues 301 and 319, and this induces osteoblast differentiation through the activation of the mitogen-activated protein kinase (MAPK) pathway. On the other hand, phosphorylation at serine residues 104 and 451 constitutes a negative effect [25]. At least ten lysine residues for the acetylation of Runx2 have been identified, thus constituting un upregulating event for Runx2 [25,26]. Methylation-affecting Runx2 activity occurs mainly through the respective enzymes at the H3K27 and H3K9 histone residues. The demethylation of H3K27me3 promotes osteoblast differentiation, whereas the methylation of H3K9 prevents Runx2 binding to promoter regions [27,28]. These modifications ultimately define the transcriptional activating or suppressing role of the Runx2 protein [25]. *Runx2* transcription is modulated by P1 and P2 promoters, while the protein exists as an heterodimer with the core-binding factor subunit beta (Cbfb), which facilitates binding to specific DNA sequences [23]. Non-coding RNAs also constitute a critical factor of bone homeostasis and Runx2 regulation. MicroRNAs (miRNAs) modulate osteoblast differentiation since a panel of miRNAs has been recognized to indirectly target Runx2 through coactivators or corepressors (Table 1). This leads to the negative or positive regulation of osteoblast differentiation, respectively [29]. Long non-coding RNAs (lncRNAs) have been also found to interact with Runx2, thereby leading to the hypertrophy of chondrocytes [30].

Runx2 is a chief driver for the maturation process of bone-specific cells. However, it is essential to note that its role extends beyond a singular function. Runx2 is tightly regulated by positive and negative feedback loops that constitute transcriptional/post-transcriptional regulatory networks. MiRNAs play a pivotal role in these regulatory circuits. Runx2 induces the transcription of miRNAs, and there is also a panel of miRNAs that post-transcriptionally modulates Runx2 activity [56]. BMP-2 induces the transcription of miR-3960, which targets homeobox A2 (Hoxa2) and upregulates Runx2 activity, whereas Runx2 also induces the transcription of miR-3960/miR2861. This mechanism constitutes an established activation/deactivation feedback loop of Runx2 [52]. The inhibitory miR-23a cluster, miR-665, and miR-31 are inhibited by Runx2. On the other hand, activating miR-3960, miR-2861, miR-302a. and miR-690 are positively regulated by Runx2 [56].

Initially, Runx2 is critical for chondrocyte differentiation, which are the cells that form the cartilage tissue. Runx2 upregulation is required for the differentiation of prehypertrophic chondrocytes into hypertrophic chondrocytes. While Runx2 role is crucial, Runx3 also plays an additional role during chondrocyte differentiation. This means that, while Runx2 is the chief driver of chondrocytes differentiation, the implication of Runx3 suggests that a more complex regulatory network governs the process. Runx2 specifically regulates the expression of Indian hedgehog homolog (IHH), a protein of the hedgehog signaling pathway and, thereby, controls the proliferation of chondrocytes. IHH can upregulate the expression of the PTH-like hormone (Pthlh), which then inhibits Runx2 expression, thus forming a negative feedback loop [57,58,59]. IHH also upregulates Runx2 expression and osteoblastogenesis in the perichondrium, an upper layer of cartilage tissue. Therefore, IHH is a Runx2-targeted protein that also presents either upregulating or downregulating effects on Runx2 activity, depending on the type and location of bone cells. In the terminal hypertrophic chondrocytes, Runx2 upregulates the angiogenic vascular endothelial growth factor A (VEGFA), secreted phosphoprotein 1 (Spp1), integrin binding sialoprotein (Ibsp), and matrix metallopeptidase 13 (Mmp13). Finally, it regulates the process through which most terminal hypertrophic chondrocytes, a type of cells during endochondral bone formation also implicated in the pathogenesis of osteoarthritis, transform into osteoblasts [60,61].

Moreover, Runx2 plays a multifaceted role during osteoblast differentiation. Following the process of the differentiation of osteoblast-lineage cells, Runx2 controls the differentiation of multipotent mesenchymal cells into pre-osteoblasts in the endochondral bone. Runx2 induces the proliferation of osteoblast progenitor cells through the induction of the hedgehog, Sp7, fibroblast growth factor (FGF), Wnt, and Pthlh signaling pathway genes. The Runx2 reciprocal induction with Wnt and Sp7 pathways promotes the differentiation of pre-osteoblasts into immature osteoblasts. Lastly, Runx2 promotes the production of bone matrix proteins in immature osteoblasts for full osteoblast maturation [60].

Runx2 is a mechano-induced bone-specific transcription factor that is subjected to dynamic activation. The ECM can have an altered degree of stiffness depending on tissue and cellular status. The rigidity and composition of the ECM is a major mechanical stimulus during bone regeneration. The data reveal that the ECM-increased stiffness upregulates Runx2 in a dose-dependent manner in mouse pre-osteoblast cells, while there is no similar effect during fibroblast differentiation [62]. ECM stiffness is also able to induce the osteogenic differentiation of human mesenchymal stem cells by upregulating Runx2 through integrin alpha-5 (α5). Integrin α5 belongs to the integrin family of receptors that mediate the communication of the ECM with cytoskeletal proteins. This event is followed by the concomitant activation of downstream mechanotransduction molecules, such as focal adhesion kinase (FAK), extracellular signal-regulated kinase (ERK), Akt, glycogen synthase kinase-3 beta (GSK-3*β*), and *β*-catenin [63]. Furthermore, shear stress, generated from fluid mechanical loading, upregulates Runx2 through the Piezo1 mechanosensitive ion channel and via the activation of the Akt/GSK-3*β*/*β*-catenin pathway in MC3T3-E1 mouse osteoblasts [64]. Piezo1 also regulates Runx2 and bone regeneration under orthodontic tooth movement in periodontal ligament (PDL) cells [65]. Protein kinase D1 (PKD1) emerges as a critical molecule for Runx2 regulation during osteoblast differentiation since the knockout of *PKD1* in vivo leads to severe deficient clinical manifestations of bone formation. This pathogenetic mechanism occurs through the signal transducer and activator of the transcription 3 (STAT3) transcription factor and p38 kinase [66].

It has been shown that mechanotransduction operates during the early phases of metastasis and promotes migration and invasion under a stiffer microenvironment. Additionally, it is under investigation how cancer cells retain this aggressive potential when they migrate to soft microenvironments, such as to the perisinusoidal bone marrow. In vivo and ex vivo experiments in breast cancer models demonstrate that Runx2 is the key driver that makes cancer cells able to preserve their acquired mechanical properties from the site of the primary tumor to the metastatic sites of softer microenvironments. Runx2 can maintain the mechanical conditioning of breast cancer cells in softer matrices, a phenomenon that has not been observed with another critical mechanosensitive transcription factor, YAP. This mechanism of the mechanical preservation of transcriptional programing represents an alternative mechanism for cancer cells to maintain their phenotype beyond genetic mutations [67].

Runx2 and the corresponding regulation of bone formation are also affected by mechano-induced mechanisms that involve autophagy. Autophagy is a highly conserved property of eukaryotic cells that involves the recycling of cellular components and ensures cellular maintenance. Following mechanical stress, mice lacking the *autophagy-related 7* (*ATG7*) gene have lower levels of both Runx2 phosphorylation and Runx2 basal expression. When autophagy is attenuated in wild-type mice cells, Runx2 phosphorylation decreases. It seems that the inhibition of autophagy and application of mechanical loading have additive effects on Runx2 downregulation, a concomitant decrease in bone formation, the suppression of ATP release, and extracellular signal-regulated kinase (ERK) downregulation [68]. In this context, mechanical stretch can also upregulate Runx2 and other osteogenic-associated biomarkers through the activation of the mechanistic target of rapamycin (mTOR) and nuclear factor kappa light-chain enhancer of activated B cells (NF-κB) in osteoblast-like cells [69]. In summary, Runx2 integrates a network of mechanotransduction pathways that mediate both bone homeostasis and related disorders.

## 4. Mechanosensitive Polycystins and their Interaction with Runx2

Polycystins constitute a distinct protein family, whose members have been acknowledged as regulators of mechanotransduction. Polycystin-1 (PC1) and polycystin-2 (PC2) are the main representatives of the polycystin family of proteins. They are encoded by the *polycystic kidney disease 1 (PKD1)* and *polycystic kidney disease 2 (PKD2)* genes, respectively. PC1 is a large protein, with approximately 4303 amino acids and a long extracellular N-terminal region, 11 transmembrane domains, and a 200-amino acid intracellular C-terminal tail (CTT). PC1 functions as an atypical G protein-coupled receptor (GPCR) and exploits its extracellular and pliable N-terminal branch to sense mechanical cues from the extracellular microenvironment [70]. PC1 interacts with G proteins, FAK, Wnt signaling, and ERK. PC1 also produces C-terminal cleavage peptides that interact with p100 subunit/signal transducer and the activator of transcription 6 (STAT6), the calcineurin/nuclear factor of activated T cells (NFAT), the regulator of G protein signaling 7 (RGS7)/14-3-3, protein kinase C (PKC), c-Jun-N-terminal kinase (JNK), the activator protein-1 (AP-1), and the Janus kinase (JAK)/STAT signaling pathways [71,72]. PC2 forms heterotetramers with PC1, thereby functioning as a selective calcium ion channel [73]. PC1 and PC2 form complexes at the plasma membrane and at immotile membrane protrusions (primary cilia), where they participate in a wider mechanosensitive structure.

**Figure 2:**
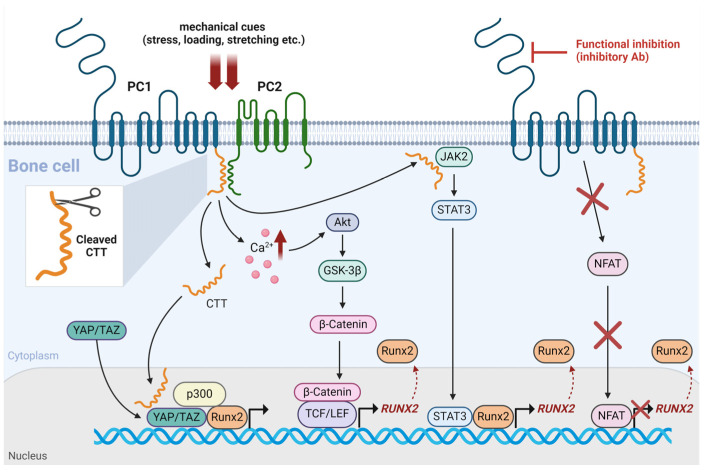
Schematic representation of polycystins and Runx2 interaction in bone mechanotransduction. CTT, C-terminal tail; GSK-3*β*, glycogen synthase kinase-3 beta; JAK2, Janus kinase 2; NFAT, nuclear factor of activated T cells; PC1, polycystin-1; PC2, polycystin-2; STAT3, signal transducer and activator of transcription 3; TAZ, transcriptional coactivator with PDZ-binding motif; TCF/LEF, T-cell factor/lymphoid enhancer fact; YAP, yes-associated protein. This figure was created using the tools provided by BioRender.com (accessed on 24 April 2024).

PC1 and PC2 are expressed in a variety of tissues and they have been implicated in a spectrum of pathophysiologies, such as during the development and progression of solid tumors [74,75,76,77,78]. Polycystins are also expressed in osteoblasts and osteocytes. It was established over twenty years ago that the loss of *PKD1* causes renal cyst development, but also defects of skeletogenesis [79]. PC1 and PC2 form heterotetrameric complexes in bone cells to respond to mechanical stress and regulate bone mass [10]. Mechanistically, PC1 CTT translocates to the nucleus and becomes physically engaged with the mechano-induced transcriptional coactivator TAZ. This interaction potentiates the association of TAZ with Runx2 and the concomitant recruitment of p300 transcriptional regulator [80]. The investigation of the PC1/TAZ interaction in mouse bone suggests that PC1 and TAZ exhibit synergic activity regarding the preservation of bone mass and anabolic bone response to mechanical loading in vivo [81]. PC1 CTT can further upregulate Runx2 expression by triggering the JAK2/STAT3 signaling axis under mechanical stretching [82]. PC1 also mediates a cellular response to mechanical tension through the induction of intracellular calcium and downstream Akt/*β*-catenin signaling pathway [83]. The functional inhibition of PC1 in PDL cells under mechanical loading causes the decrease in Runx2 expression and the reduction in the activated form of its upstream regulator, NFATc1 [84]. These observations suggest that PC1 mediates the impact of Runx2 on osteoblast differentiation and bone formation by functioning synergically with TAZ and through different signaling pathways (Figure 2).

For an efficient building of bone structure, bone cells trigger a self-assembly process regulated by the bone genetic regulatory network (GRN). This network is activated by the canonical pathway of bone formation, which involves the Wnt/*β*-catenin canonical axis. Stiffness induces Wnt/*β*-catenin to ensure the process of bone mineralization through mechanotransduction, and PC1 may act through this pathway as a mechanoreceptor [85]. Gene expression experiments have demonstrated the direct association of polycystins with bone-specific regulators. The loss of *PKD1* is accompanied by bone loss defects in mature tissues. This *PKD1* loss of expression in osteoblasts in vivo causes a gene dose-dependent reduction in bone mineral density, trabecular bone volume, cortical thickness, and the expression of osteoblast-specific genes, such as *runx2*, *osteocalcin*, *osteopontin*, and *bone sialoprotein* [86]. Homozygous inactivating mutations of *PKD1* are followed by delayed bone formation, whereas heterozygous inactivation results in the problematic function of osteoblasts and osteopenia. These features are associated with a diminished expression of Runx2 [87]. Apart from osteoblasts, PC1 functions as a mechanosensor in osteocytes and mediates the anabolic impact of mechanical loading on bone regeneration. When *PKD1* is deleted, the application of fluid shear stress is not able to evoke bone formation in vivo [88]. On the other hand, the in vitro knockdown of *PKD1* in osteoblasts can lead to the increased proliferation and impaired differentiation of and concomitant enhancement in adipogenesis via the cyclic adenosine monophosphate (cAMP)/protein kinase A (PKA) pathway [89]. Furthermore, the intact function of PC1 is required for the proper proliferation of cranial osteochondroprogenitor cells and the development of cranial sutures and synchondroses [90]. PC1 affects the expression of Runx2 and osteocalcin in craniosynostosis in vitro, whereas the functional inhibition of PC1 in primary cranial suture cells increases proliferation and migration and activates the Akt/mTORC2 pathway [91,92]. Mechanistically, PC1, as a mechanoreceptor, probably senses mechanical loading, such as stiffness, and initiates bone mineralization via the mechanisms of mechanotransduction [85]. These findings lead to the conclusion that PC1’s proper function and intact expression are prerequisites for the normal regulation of bone homeostasis by mechanical loading.

The loss of *PKD2* gene expression suppresses bone mass, mechanotransduction properties, the YAP/TAZ expression of osteoblast-specific genes in vivo, and Runx2 in bone ex vivo. Opposite to *PKD1*, the loss of *PKD2* expression reduces peroxisome proliferator-activated receptor gamma (PPAR*γ*), the deposition of bone marrow fat, and adipogenesis in vivo [93]. *PKD2* loss-of-function mutation induces several craniofacial abnormalities, which are also detected in autosomal dominant polycystic kidney disease (ADPKD) patients [94]. PC1 and PC2 can both interact in a complex with TAZ to induce osteoblastogenesis and suppress adipogenesis [95]. PC2 specifically potentiates the osteogenic differentiation of human adipose-derived stem cells through TAZ-dependent Runx2 upregulation [96]. Therefore, PC2 normal function is also needed for the homeostasis of bone cells, and the PC1/PC2/TAZ/Runx2 complex presents an appealing therapeutic target for age-related osteoporosis.

## 5. Therapeutic Opportunities

Mechanotransduction is a distinct discipline in the field of biomechanical engineering, and it seems promising for the development of novel therapies of bone tissue disorders, such as osteoporosis. In this regard, Runx2 and polycystins emerge in mutual signaling networks, thereby offering potential treatment options. A recent study shows that the PC1/PC2 complex, harbored in the primary cilia of osteoblasts, has been detected to transduce signals that regulate bone mass as a response to hypoxia exposure. In hypoxic conditions, hypoxia-inducible factor alpha (HIF-1α) translocation and accumulation to the nucleus promotes elevated bone mass with VEGF-associated increased vascularization, the upregulation of anaerobic glycolysis, and glucose uptake. However, an increased conversion of glucose to lactate and increased glucose uptake are also observed under normoxia conditions in cancer cells, constituting the well-known Warburg effect. This polycystin-associated mechanism elucidates how hypoxia, present at high altitudes, has a diminishing effect on bone homeostasis. It also represents a potential therapeutic target since the application of pulsed electromagnetic field could suppress bone loss through the primary cilium/PC1/PC2/HIF-1α cascade [97]. On the other hand, Runx2 has already been evaluated as a biomarker of bone regeneration in novel biomechanical approaches. In cases of bone loss, a new method encompassing targeted magnetic stimulus evokes mechanotransduction and the induction of Runx2 and alkaline phosphatase to foster osteogenic differentiation with a drug-free process [98]. An additional magnetic nanoparticle (MNP)-based platform can remotely apply mechanical stimulus to bind to integrin-α5 and upregulate Runx2 and lymphoid enhancer-binding factor 1 (LEF1). Such mechanism represents an alternative, non-antibody-mediating strategy for bone regeneration [99]. A different biomaterial that holds promise for clinical application, the nanoparticulate mineralized collagen glycosaminoglycan (MC-GAG) scaffold, demonstrates a substrate-dependent impact on osteogenic differentiation in primary human bone marrow-derived mesenchymal stem cells. The increased stiffness of these scaffolds induces the colocalization of YAP and *β*-catenin. The downregulation of *β*-catenin induces Runx2 expression only on stiff materials, and *β*-catenin functions as a negative modulator of osteogenic differentiation [100]. Moreover, electrical stimulus through bioactive glass nanoparticles exhibits a promising activity of provoking the differentiation of bone cells through the bone morphogenetic protein (BMP)/Smad4 pathway and the corresponding *runx2*/*osteocalcin* transcription in primary bone marrow-derived human mesenchymal stem cells [101].

MiRNAs encompass a large family of epigenetic regulators, which are implicated in therapeutic strategies and are also linked to the modulation of Runx2 and polycystins’ activity (Table 1). There is a panel of miRNAs that target Runx2 and, therefore, represent potential tools for therapeutic utility through the administration of anti-miRNAs and miRNA mimics [29]. The data also highlight the role of mechanosensing lncRNAs, such as nuclear enriched abundant transcript 1 (Neat1). *Neat1* knockout mice display impaired bone formation and reduced bone mass. Mechanical loading is not able to induce bone regeneration in *Neat1* knockout mice. Mechanistically, the lack of a response to the mechanical loading of *Neat1*-deficient osteoblasts occurs through Smad ubiquitination regulatory factor 1 (Smurf1)/Runx2 targeting [102]. Tensile strain is also a type of dynamic induction of osteogenic differentiation. This type of strain can enhance osteogenesis from bone marrow mesenchymal stem cells by upregulating lncRNA maternally expressed 3 (MEG3), which, in turn, downregulates miR-140-5p and causes Runx2-mediated differentiation [103].

Genetic defects of *Runx2* or defects on pathways concerning post-translational modifications of Runx2 cause craniofacial malformations. Furthermore, the aberrant upregulation of fibroblast growth factor receptors (FGFRs) is implicated in the pathophysiology of craniosynostosis through Runx2, having as clinical manifestations the premature fusion of sutures, the concomitant deformation of the skull, and facial hypoplasia. The inhibition of this upregulation is a promising therapeutic target. Polycystins have been also implicated in craniosynostosis [91,104]. Therefore, the combinatorial targeting of Runx2 and polycystins could provide an additional therapeutic outcome.

Although there is a panel of targeting drugs that have been FDA-approved for bone diseases, currently, bone remodeling biology has not been exploited in the clinic. In this context, the current settings of therapeutic efforts regarding bone disorders involve mechanotransduction molecules and drug delivery systems that are being tested in several pathophysiologies. These efforts involve specific gene targeting or protein expression to control protein–protein interactions and enhance bone formation against bone resorption [105]. For example, the targeted inhibition of integrin *α*5*β*3 reverses bone loss and increases bone mineral density in women with osteoporosis following menopause [106]. Phase I clinical study targeting *α*5*β*1 integrin has been also completed for patients with osteoarthritis who are destined for total knee replacement (NCT02491281). Patients were injected intra-articularly with the LNA043 agent. Transcriptomics profiling reveal the repression of osteoarthritis progression, the induction of anabolic pathways, and chondrogenesis [107]. Other integrins have been also found to be promising in preclinical studies; however, there are challenges due to their extensive participation in biological processes and other side effects.

## 6. Conclusions

It has been established that the biomechanical features of bone defects and disorders offer novel therapeutic strategies apart from drug-associated treatments. In this vein, the multifactorial role of Runx2 provides a vast array of potential targets that are being preclinically and clinically evaluated. Runx2 plays a decisive role in orchestrating osteoblast differentiation, chondrocyte maturation, and response to mechanical cues, thus defining crucial mechanisms in bone physiology and pathophysiology. Runx2 is a critical bone-specific factor, but it seems that more complex signaling circuits remain to be elucidated. To this end, PC1 and PC2 emerge as an upstream mechanosensitive protein complex, which navigates most of the bone-associated signaling pathways following mechanical stimulation. Polycystins impact bone physiology through intricate interactions with signaling pathways, such as Runx2 and YAP/TAZ, thereby influencing osteoblast differentiation and bone formation. Both Runx2 and the polycystin family of proteins emerge as key players in mutual signaling networks, offering potential treatment options through various mechanisms, such as the modulation of hypoxia-induced pathways, targeted magnetic stimulation, nanoparticle-based platforms, and epigenetic regulation via miRNAs and lncRNAs. Because PC1, in particular, presents challenges and extensive troubleshooting as an experimental task, progress has to be made towards this direction. On the other hand, different types of bone cells are subjected to a vast array of mechanical inputs, which is difficult to simulate in vivo. To map the complex and cross-talking regulatory signaling networks, it is necessary to generate experimental data from more sophisticated experimental models, using 3-dimensional cell cultures and novel substrate materials. Furthermore, studies should also incorporate devices to simulate more accurately the physical microenvironment of the cells. Future research efforts will shed light into the detailed role of this signaling circuitry and will potentially incorporate these key elements in selective treatment approaches.

## Figures and Tables

**Table 1:** List of microRNAs (miRNAs) modulating the expression of polycystins and Runx2.

MicroRNA	Mechanism	Tissue	Reference
miR-17	*Polycystic Kidney Disease 1 (PKD1)* and *Polycystic Kidney Disease 2 (PKD2)* mRNAs suppressed via their 3’-UTR miR-17-binding motif Targets *PKD2*	Autosomal dominant polycystic kidney disease (ADPKD) mouse models HEK 293T	[31,32]
miR-17∼92	Upregulated and produces renal cysts in mice	Polycystic kidney disease (PKD) mouse models	[33]
miR-200 family (miR-200b, miR-200c, and miR-429)	Downregulation of *PKD1* by miRNAs secreted by exosomes Regulated by hepatocyte nuclear factor-1 (HNF-1) and targets *PKD1* miR-200 downregulation by Dicer inactivation and concomitant *PKD1* upregulation	Exosomes secreted from cystic epithelial cells and urine exosomes	[34,35,36]
miR-4787-5p	Targets *PKD1* mRNA	Vascular smooth muscle cells (aortic dissection)	[37,38]
miR-17, miR-200c, and miR-182	p68 induces the expression of miR-17, miR-200c, and miR-182 targeting *PKD1* mRNA	Renal epithelial cells	[39]
lncRNAs OIP5-AS1 and UGDH-AS1	Target *PKD1*	Bioinformatics for intervertebral disc degeneration	[40]
miR-181a	Targets B-cell lymphoma 2 (Bcl-2) and promotes PKD phenotype	Plasma of PKD patient	[41]
miR-106b-5p	Targets *PKD2* and sensitizes A549 cells to cisplatin	Non-small-cell lung cancer	[42]
miR-199a-5p	Targets Runx2 through SMAD1/5/9	Human mesenchymal stem/progenitor cells	[43]
circ_0076684/miR-370-3p, miR-140-3p, and miR-193a-5p	CBX4-mediated Runx2 and circ_0076684 upregulation	Osteosarcoma	[44]
miR-338-3p	Targets Runx2 from upstream circ-3626 regulation	Mouse bone marrow-derived mesenchymal stem cells	[45]
miR-584-5p	Hypoxia-induced osteogenic differentiation through Runx2	Primary mouse periosteal stem cells	[46]
miR-205-5p	Targets Runx2 by upstream circ-FK501 binding protein 51 induction	Bone marrow mesenchymal stem cells	[47]
miR-224-5p	Targets Runx2 for osteoblast differentiation	C2C12 myoblast cells	
miR-300	Osteoblast differentiation through Smad3/*β*-catenin/RunX2	Primary rat osteoblast cells, human osteoblast culture	[48]
miR-23b	Targets Runx2 induced by bone morphogenetic protein 9	C2C12 myoblast cells	[49]
miR-21	Osteogenic differentiation through Smad7-Smad1/5/8-Runx2	Bone marrow mesenchymal stem cells	[50]
miR-505	Osteogenic differentiation through Runx2	MC3T3-E1 cells	[51]
miR-3960/miR2861	BMP-2/miR-3960/homeobox A2 (Hoxa2) Runx2/miR-3960/miR2861 feedback loop	Primary mouse calvarial osteoblasts	[52]
miR-467g	Osteoblast differntiation through IHH/Runx2 targeting	Primary mouse calvarial osteoblasts,	[53]
		human mesenchymal (skeletal) stem cells	
miR-204	Targets Runx2 promoting adipocyte differentiation	Mesenchymal progenitor cells and bone marrow stromal cells	[54]
miR-23a, miR-30c, miR-34c, miR-133a, miR-135a, miR-137, miR-204, miR-205, miR-217, and miR-338	Target Runx2 suppressing osteoblast differentiation	Mouse MC3T3-E1 osteoblasts, Mouse ATDC5 chondrocytes	[55]

## Data Availability

Not applicable.
